# Standardizing Single-Frame Phase Singularity Identification Algorithms and Parameters in Phase Mapping During Human Atrial Fibrillation

**DOI:** 10.3389/fphys.2020.00869

**Published:** 2020-07-21

**Authors:** Xin Li, Tiago P. Almeida, Nawshin Dastagir, María S. Guillem, João Salinet, Gavin S. Chu, Peter J. Stafford, Fernando S. Schlindwein, G. André Ng

**Affiliations:** ^1^Department of Cardiovascular Science, University of Leicester, Leicester, United Kingdom; ^2^School of Engineering, University of Leicester, Leicester, United Kingdom; ^3^Aeronautics Institute of Technology, ITA, São José dos Campos, Brazil; ^4^Auckland Bioengineering Institute, University of Auckland, Auckland, New Zealand; ^5^Universitat Politècnica de València, Valencia, Spain; ^6^Centre for Engineering, Modelling and Applied Social Sciences, Federal University of ABC, Santo André, Brazil; ^7^National Institute for Health Research Leicester Cardiovascular Biomedical Research Centre, Glenfield Hospital, Leicester, United Kingdom

**Keywords:** atrial fibrillation, catheter ablation, non-contact mapping, atrial electrograms, phase singularity, rotor, spiral wave

## Abstract

**Purpose:**

Recent investigations failed to reproduce the positive rotor-guided ablation outcomes shown by initial studies for treating persistent atrial fibrillation (persAF). Phase singularity (PS) is an important feature for AF driver detection, but algorithms for automated PS identification differ. We aim to investigate the performance of four different techniques for automated PS detection.

**Methods:**

2048-channel virtual electrogram (VEGM) and electrocardiogram signals were collected for 30 s from 10 patients undergoing persAF ablation. QRST-subtraction was performed and VEGMs were processed using sinusoidal wavelet reconstruction. The phase was obtained using Hilbert transform. PSs were detected using four algorithms: (1) 2D image processing based and neighbor-indexing algorithm; (2) 3D neighbor-indexing algorithm; (3) 2D kernel convolutional algorithm estimating topological charge; (4) topological charge estimation on 3D mesh. PS annotations were compared using the structural similarity index (SSIM) and Pearson’s correlation coefficient (CORR). Optimized parameters to improve detection accuracy were found for all four algorithms using *F*_β_ score and 10-fold cross-validation compared with manual annotation. Local clustering with density-based spatial clustering of applications with noise (DBSCAN) was proposed to improve algorithms 3 and 4.

**Results:**

The PS density maps created by each algorithm with default parameters were poorly correlated. Phase gradient threshold and search radius (or kernels) were shown to affect PS detections. The processing times for the algorithms were significantly different (*p* < 0.0001). The *F*_β_ scores for algorithms 1, 2, 3, 3 + DBSCAN, 4 and 4 + DBSCAN were 0.547, 0.645, 0.742, 0.828, 0.656, and 0.831. Algorithm 4 + DBSCAN achieved the best classification performance with acceptable processing time (2.0 ± 0.3 s).

**Conclusion:**

AF driver identification is dependent on the PS detection algorithms and their parameters, which could explain some of the inconsistencies in rotor-guided ablation outcomes in different studies. For 3D triangulated meshes, algorithm 4 + DBSCAN with optimal parameters was the best solution for real-time, automated PS detection due to accuracy and speed. Similarly, algorithm 3 + DBSCAN with optimal parameters is preferred for uniform 2D meshes. Such algorithms – and parameters – should be preferred in future clinical studies for identifying AF drivers and minimizing methodological heterogeneities. This would facilitate comparisons in rotor-guided ablation outcomes in future works.

## Introduction

Atrial fibrillation (AF) is the most common cardiac arrhythmia in clinical practice, affecting 1–2% of the worldwide population (Nattel, 2002). AF increases 5-fold the risk of stroke and is related with increased mortality and significant high costs in medical treatments (Nattel, 2002). Although catheter ablation has been shown effective in treating paroxysmal AF, the identification of areas for successful ablation in patients with persistent AF (persAF) remains challenging due to the possible existence of multiple arrhythmogenic mechanisms (Nattel, 2003; Guillem et al., 2016). Recently, the localized sources and rotors theory has gained evidence to explain sustained fibrillatory behavior during AF (Pertsov et al., 1993; Jalife et al., 2002; Pandit and Jalife, 2013). Early data have shown ablation of localized sources to be useful to eliminate AF (Narayan et al., 2012b, 2014; Schricker et al., 2014), but subsequent works have failed to reproduce such results, which motivated intense debate on the efficacy of rotor-guided ablation as a therapy for persAF (Benharash et al., 2015; Jalife et al., 2015).

**FIGURE 1:**
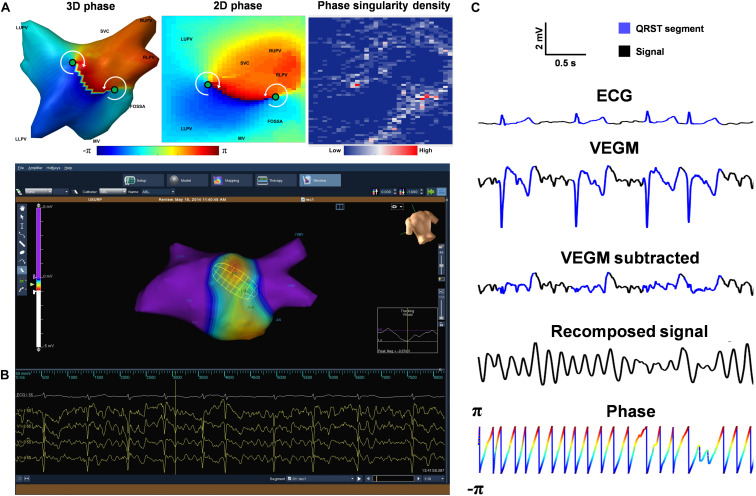
Data acquisition and signal processing. **(A)** Reconstructed 3D left atrial geometry with color-coded phase map, its 2D representation (cylinder projection) showing PS points (green circles) and example of a 2D PSD map. **(B)** The screenshot of the Ensite Velocity mapping system showing a isopotential/voltage map with the non-contact Ensite Array catheter. **(C)** Example of ECG (Lead I), VEGM, QRST-subtracted VEGM, recomposed signal using sinusoidal wavelet reconstruction and Phase signal (color-coded by phase), with the QRST segments highlighted in blue. LUPV, Left Upper Pulmonary Vein; RUPV, Right Upper Pulmonary Vein; LLPV, Left Lower Pulmonary Vein; SVC, Superior vena cava; MV, Mitral valve.

Phase mapping has become broadly accepted to map rotors in AF since it facilitates the visualization of the underlying dynamics and spatiotemporal behavior of cardiac activations (Umapathy et al., 2010; Kuklik et al., 2015, 2017; Roney et al., 2017). Phase singularity (PS) – found at the tip of a rotor – is a key feature for the location and tracking of such rotational activities (Umapathy et al., 2010). Therefore, the analysis of PS dynamics is important for understanding the mechanisms of the arrhythmia (Salinet et al., 2017). As illustrated in Figure 1A, PS is generally defined as the point – in a single phase map – around which the phase progresses monotonically through a complete 2π cycle (Gray et al., 1998; Umapathy et al., 2010; Guillem et al., 2013). During automated PS detection, it is common that (i) a phase threshold is used to facilitate the detection of phase gradients – usually slightly lower than a full 2π rotation around the point of interest and; (ii) a search radius is considered to define the most distant neighboring node used by the algorithm for assessing phase gradients (Roney et al., 2017).

Different techniques for automated PS detection have been proposed and have been broadly used in electrophysiological (EP) studies, each of which considering different aspects and characteristics of the phase map (Bray et al., 2001; Rantner et al., 2007; Tomii et al., 2015). In 2001, Bray et al. (2001) developed a “topological charge” method for PS detection, based on convolutional kernels which became one of the most popular methods for PS detection. Iyer and Gray suggested a shorter path length may give a more precise localization but may miss phase singularities (Iyer and Gray, 2001). Different convolutional kernels which modify the path length for the topological charge integral have been used (Bray et al., 2001; Bray and Wikswo, 2002b; Zhuchkova and Clayton, 2005), but the effect of using different kernels has not been investigated. Rantner et al. (2007) developed a topological charge solution that can be used on 3D triangular meshes. These methodologies – based on different criteria – might culminate in distinct detected PSs, subsequently affecting AF driver identification, which could partially explain the recent inconsistencies in rotor-guided ablation outcomes (Benharash et al., 2015; Buch et al., 2016; Steinberg et al., 2017; Gianni et al., 2016). Finally, the absence of investigations regarding the details of different methodologies used for automated PS identification and their spatiotemporal behavior makes the comparison among studies – and assumptions about the arrhythmia – difficult. Therefore, the quantitative analysis of the underlying fibrillatory activations based on dynamic phase mapping remains a challenge (Salinet et al., 2017). In this study, we aim to investigate the performance of four different techniques for automated PS detection and the effect of two important parameters – the phase gradient threshold and the search radius – using non-contact mapping (NCM) in human persAF.

## Materials and Methods

### Electrophysiological Study

The present study was approved by the local ethics committee for patients undergoing AF ablation at the University Hospitals of Leicester NHS Trust. Ten patients undergoing catheter ablation of persAF for the first time were recruited for the USURP-AF (Understanding the electrophysiological SUbstRate of Persistent Atrial Fibrillation) study. The details of the patients’ baseline characteristics are presented in Supplementary Figure S1.

Prior to the EP study, all drugs except amiodarone were stopped for at least four half-lives. Bilateral femoral venous access was achieved under fluoroscopic guidance, and a quadripolar catheter and a deflectable decapolar catheter were placed at the His position and Coronary Sinus (CS), respectively. Trans-septal puncture was performed to gain access to the left atrium (LA). A non-contact multi-electrode array (MEA) catheter (EnSite Velocity, St. Jude Medical, United States) and a conventional deflectable mapping catheter were deployed in the LA. Anticoagulant drugs were administered to maintain an activated clotting time >300 s. A high-resolution 3D LA geometry was created using EnSite Velocity electro-anatomical mapping system (St Jude Medical, now Abbott) and anatomical locations were annotated (Figure 1B). No rotors were ablated in this protocol.

### Left Atrial Geometry and Virtual Electrogram

The non-contact MEA catheter from EnSite Velocity has 64 electrodes. The EnSite system employs an inverse solution to estimate the potentials on the endocardium. The potentials from the 64 electrodes on the MEA are used to estimate virtual electrograms (VEGMs) in 64 locations on the endocardium, which are further interpolated to provide a total of 2048 VEGMs. The 3D vertices corresponding to the locations of the 2048 VEGMs were exported from the mapping system and triangulated to a 3D mesh for each patient. The 2048 locations on the 3D shell are organized by the EnSite system in the same way as the “map projection” of the globe, where there are 64 “longitude lines” and 32 “latitude lines” with the intersecting points being the 2048 vertices. Therefore, this setting provides a natural point-by-point cylindrical projection when opening the 3D mesh to a 2D rectangular mesh (64 × 32), which does not induce additional distortions.

### Data Acquisition and Signal Processing

2048 baseline VEGMs and surface electrocardiogram were collected with a sampling frequency of 2034.5 Hz (Figure 1C). The signals were band-pass filtered (1–150 Hz) by the Ensite system with default setting, exported and analyzed offline using Matlab (Mathworks, MA, United States, version 2018a). For each patient, 30 s of VEGMs were resampled to 512 Hz using a cubic spline interpolation to reduce processing time. Downsampling the electrograms to 512 Hz does not result in loss of information in the VEGMs, as the signals were sampled at a relatively high frequency. The down sampled version is still comfortably within the Nyquist criterion – considering the frequency content with relevant electrophysiologic information (1–150 Hz) – and allows the capture of details of even the fastest physiological fluctuations (Stevenson and Soejima, 2005). Ventricular far-field activity was removed from the recorded VEGMs using a QRST subtraction technique previously described (Figure 1C; Salinet et al., 2013a).

### VEGM Pre-processing

The wavelet/sinusoidal reconstruction proposed by Kuklik et al. (2017) is commonly used in intracardiac signals to unveil the underlying wavefront propagation and investigate re-entry circuits. Accordingly, the local atrial cycle length (in seconds) is used as an input for the wavelet/sinusoidal reconstruction. In the present work, the local atrial cycle length was calculated as the inverse of the dominant frequency (DF, in hertz) for each VEGM. The reconstructed VEGMs were then used for the phase calculation (Figure 1C).

### Phase Mapping

Hilbert transform *h*(*t*) of the reconstructed VEGMs *f*(*t*) was used to generate an analytic signal *F*(*t*), from which the instantaneous phase φ(*t*) of the VEGMs was obtained as the four-quadrant inverse tangent (function *atan2* in MATLAB) of the ratio of the imaginary*h*(*t*) and real part *f*(*t*) of the analytic signal (Eq. 1, Figure 1C; Umapathy et al., 2010; Clayton and Nash, 2015; Ortigosa et al., 2015).

$$F\,\left(t\right)=f\,\left(t\right)+j\,\,h\,\left(t\right)=A\,\left(t\right)\,\,e^{j\,\,\varphi \,\left(t\right)}$$

(1) φ⁢(t)=a⁢t⁢a⁢n⁢2⁢[h⁢(t),f⁢(t)⁢]

### The Detection of Phase Singularities

**FIGURE 2:**
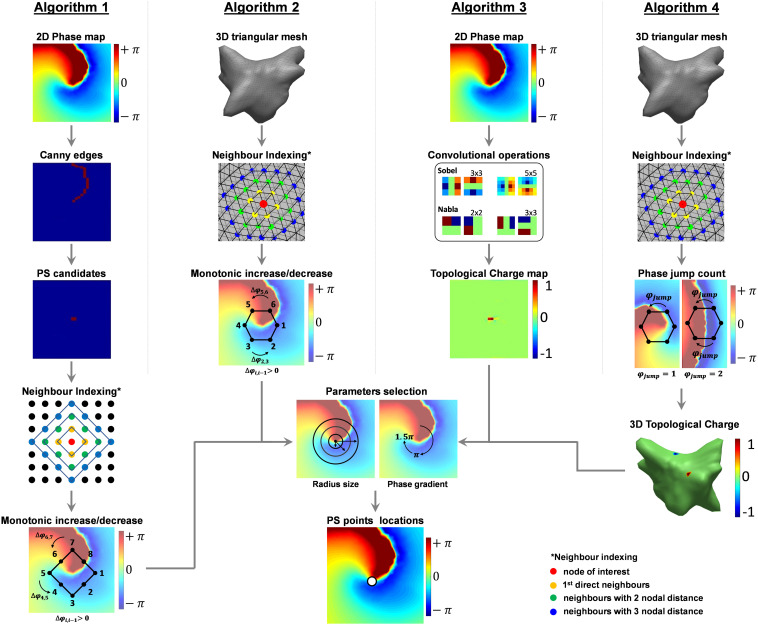
Schematic of the three algorithms of phase singularity detection. Briefly, **Algorithm 1** – Image Processing-based Algorithm: (1) Canny edge detector to locate the line with large phase gradient, (2) PS candidates pre-selected as the ends of the edge lines, (3) checking the neighbors of each candidate for monotonic change of phase, (4) applying phase gradient threshold to locate PS points; (5) Clustering PSs referring same PS using center of gravity of the cluster. **Algorithm 2** – 3D Triangulation algorithm: (1) neighbors of all nodes on the 3D mesh were indexed from triangulation, (2) checking the neighbors of each node for monotonic change of phase, (3) applying phase gradient threshold to locate PS points, and (4) clustering using DBSCAN. **Algorithm 3** – Topological charge: (1) calculating topologic charge using different kernels, and (2) applying topological charge threshold. **Algorithm 4** – Topological charge on a 3D mesh: (1) neighbors of all nodes on the 3D mesh were indexed from triangulation, and (2) count number of “phase jumps” using topological charge, (3) assigning topological charge based on the count number.

Four consolidated techniques commonly used for the automated detection of PSs were considered in the current study, as illustrated in Figure 2. The details are described in the following sections.

#### Algorithm 1 – Image Processing-Based Algorithm

Algorithm 1 was originally designed to work with 2D optical mapping (Climent et al., 2015), for applications on 2D uniform rectangular meshes. First, the 2D meshes were generated using cylindrical projection in the triangulated 3D meshes exported from the EnSite system (Salinet et al., 2013b). Sharp edges of relative large phase gradients were then detected using Canny edge detector, as illustrated in Figure 2 (Canny, 1986). Points at the ends of the edge lines were detected and selected as candidates for PSs. The neighbors around the candidates were defined as a “diamond” expansion and sorted clockwise (Figure 2, Algorithm 1), and a PS was marked if (i) a monotonic increase/decrease was detected along a loop of neighboring nodes around the node of interest and; (ii) the phase gradient within that loop of neighboring nodes [max(φ_*Loop*_)−min(φ_*Loop*_)] exceeded an operator-defined threshold. The default threshold for this algorithm is 1.5π (Climent et al., 2015).

#### Algorithm 2 – 3D Triangulation Algorithm

Algorithm 2 is an in-house algorithm developed for analyzing the triangulated 3D mesh with VEGMs. The neighbor indices of the nodes were found from the 3D triangulation mesh, and the neighbors were sorted clockwise (Figure 2, Algorithm 2). Increases or decreases of the phase of the neighbors were detected and a PS was identified if (i) a monotonic increase/decrease was detected from the sorted neighbors along a loop of neighboring nodes around the node of interest and; (ii) the phase gradient within that loop of neighboring nodes [max(φ_*Loop*_)−min(φ_*Loop*_)] exceeded an operator-defined threshold. The default threshold for this algorithm is 1.5π (Li et al., 2017a). The detections were translated into the 2D mesh using cylindrical projection.

#### Algorithm 3 – Topological Charge Algorithm

Algorithm 3 is one of the most commonly used PS detection methods by investigators, which estimate the topological charge from 2D uniform rectangular meshes. It evaluates the contour integral of the phase gradient around the nodes of interest using a sliding matrix (kernel) in the 2D space. The PSs are detected by computing the topologic charge density as the curl of the spatial phase gradient (Figure 2, Algorithm 3). Bray et al. (2001) and Bray and Wikswo (2002b) implemented this technique based on the “topologic charge” index, *n*_*t*_:

(2) $$n_{t}\equiv \underset{c}{\frac{1}{2\,\pi }}\,\nabla \phi \,\left(\vec{r}\right)\cdot d\,\vec{l}$$

where *n*_*t*_ is the topologic charge index$\varphi \,\left(\vec{r}\right)$ is the local phase, the line integral is taken over path $\vec{l}$ on a closed curve *c* surrounding the PS candidate (the region where the phase is undefined). Bray et al. (2001) also demonstrated the computation of the line integral (Eq. 3) in Eq. (2) at any location may be expressed as a 2D convolution operation using a 3 × 3 matrix of weights – i.e., a kernel – in each of the *x* and *y* directions, which allows efficient computation (Bray et al., 2001):

(3) $$l\,i\,n\,e\,i\,n\,t\,e\,g\,r\,a\,l\,\nabla_{x}⊗k_{y}+\nabla_{y}⊗k_{x}$$

Where ⊗ is the convolutional operator, *k*_*x*_ and *k*_*y*_ are the phase gradients in vertical and horizontal directions. Different convolutional kernels were used in different works (Bray et al., 2001; Bray and Wikswo, 2002b), and four kernels were included in the present study: sobel 3 × 3, sobel 5 × 5, nabla 2 × 2 and nabla 3 × 3 (Figure 2 illustrated color-coded examples of the kernels, in Algorithm 3 column). The kernels are illustrated in Supplementary Figure S1). As an example, the sobel 3 × 3 convolutional kernels (*∇*_*x*_ and *∇*_*y*_) are defined as (Eqs. 4, 5):

(4) $$\nabla_{x}=\left[\begin{array}{ccc} -1/2 & 0 & +1/2\\ -1 & 0 & +1\\ -1/2 & 0 & +1/2 \end{array}\right]$$

(5) $$\nabla_{y}=\left[\begin{array}{ccc} +1/2 & +1 & +1/2\\ 0 & 0 & 0\\ -1/2 & -1 & -1/2 \end{array}\right]$$

Similarly, Eqs. 6, 7 are an example for the nabla 2 × 2 kernel:

(6) $$\nabla_{x}=\left[\begin{array}{cc} 1 & -1\\ 0 & 0 \end{array}\right]$$

(7) $$\nabla_{y}=\left[\begin{array}{cc} -1 & 0\\ 1 & 0 \end{array}\right]$$

The default phase threshold for PS detection is 1.9π. Therefore, PSs were annotated if 2π⋅*n*_*t*_ was more negative than -1.9π or if it was higher than +1.9π – the sign being the chirality of the rotation, i.e., the direction in which the associated wave front circulates about the PS (clockwise or counter clockwise).

#### Algorithm 4 – 3D Topological Charge Algorithm

Algorithm 4 (3D topological charge algorithm) (Rantner et al., 2007) is based on the concept of estimating the topological charge as in Eq. (2) (Bray et al., 2001; Bray and Wikswo, 2002b). The neighbor index of the nodes was found from the triangulated 3D mesh, and the neighbors were sorted clockwise (Figure 2, Algorithm 4). The sorted neighbors form a closed path around the node of interest, and the radius of the path can be defined as a search parameter of N nodal distance. From this closed path, the algorithm counts the occurrence of sudden “phase jumps” from – π to π and vice-versa (Figure 2, Algorithm 4). This “phase jump,” however, is usually below 2π due to limited resolution of discrete meshes. Therefore, a “phase jump” is annotated when the difference of two neighboring nodes along the circular path exceeds a phase gradient threshold. The default threshold of this phase gradient is 3.5 (∼1.1π) (Rantner et al., 2007). As illustrated in Figure 2 (Algorithm 4), an odd number of “phase jumps” is expected at PS points, whereas even numbers suggests no PS. Topological charge of value 1 will be assigned to positive odd number counts, -1 is for negative odd number, and 0 for all even number counts – where there is no topological charge. The sign of this topological charge corresponds to the chirality of the rotation.

#### Local Cluster Refinement

In PS detection, the neighboring nodes of a detected PS may also satisfy conditions for PS annotation, resulting in a small cluster of nodes next to each other. Therefore, PS detection methods might benefit from a local cluster refinement that select one single PS as representative of such cluster.

The default version of algorithms 1 and 2 already include methods for filtering out extra detected PSs, whereas the default version of algorithms 3 and 4 consider none. Algorithm 1 adopts the center of gravity of a cluster as the representing PS, and algorithm 2 considers a modified version of the density-based spatial clustering of applications with noise (DBSCAN) (Ester et al., 1996).

DBSCAN arranges high-density points that are closely packed and rejects neighboring points that lie alone in low-density regions as outliers. Usually, a distance threshold is considered to define the neighbors. In the present work, this neighbor-searching distance threshold has been replaced by direct neighbors from a triangulation mesh. A distance threshold of 5 mm was introduced for each iteration.

Since algorithms 3 and 4 have no clustering step by default, the effect of adding clustering via DBSCAN was also included in this study. In summary, the following analyses were performed in the subsequent parts of this work: algorithm 1, algorithm 2, algorithm 3, “algorithm 3 + DBSCAN,” algorithm 4 and “algorithm 4 + DBSCAN.”

Examples of the effect of DBSCAN on removing multiple PSs referring the same location can be found in Supplementary Figure S2.

### Parameter Sensitivity

#### Phase Gradient

A set of phase gradient thresholds ranging from 0.1π to 2π were investigated and applied on all the algorithms. The phase gradient parameter was also investigated for the 2D topological charge (algorithm 3). In this case, however, the thresholds applied were an equivalent to the topological charge instead of the phase gradient.

#### Search Radius

The phase spatial diffusion was also considered in the analysis for marking a PS. Therefore, different search radii were tested, varying from 1 to 8 nodal distances from the node of interest – i.e., nodes with potential PSs – with exception for algorithm 1 that starts from 2 nodal distances.

Search radii were not investigated in algorithm 3 as it uses convolutional operators (kernels) instead of iterations of neighboring node (as in algorithms 1, 2). In order to investigate the effect of the phase spatial diffusion using algorithm 3, four different kernels were investigated: sobel 3 × 3, sobel 5 × 5, nabla 2 × 2, and nabla 3 × 3 (Supplementary Figure S1).

### Similarity Measurements

Once PSs were detected for the different parameters configurations, PS density (PSD) maps were created for the algorithms. Each PSD map was defined as a 64 × 32 matrix with each “pixel” representing the number of times that a PS has been visited (Figure 1A, PSD). The normalized PSDs were compared using two indices measuring the similarity between the PSD maps and those annotated by an expert (see “Clinical Annotation” section below): structural similarity Index (SSIM) (Zhou et al., 2004) and Pearson’s Correlation Coefficient (CORR) (Pearson, 1896).

#### Structural Similarity Index

The SSIM ranges between -1 and 1, where 1 corresponds to two identical sets of data, 0 represents no correlation and -1 represents inversed sets of data. In Eq. (8), three factors (first row) estimate similarity according to luminance, contrast and structure (Zhou et al., 2004).

$$S\,S\,I\,M=\frac{\left(2\,\mu_{x}\,\mu_{y}+c_{1}\right)}{\left(\mu_{x}^{2}+\mu_{y}^{2}+c_{1}\right)}\cdot \frac{\left(2\,\sigma_{x}\,\sigma_{y}+c_{2}\right)}{\left(\sigma_{x}^{2}+\sigma_{y}^{2}+c_{2}\right)}\cdot \frac{\left(\sigma_{x\,y}+0.5\,c_{2}\right)}{\left(\sigma_{x}\,\sigma_{y}+0.5\,c_{2}\right)}$$

(8) $$=\frac{\left(2\,\mu_{x}\,\mu_{y}+c_{1}\right)\,\left(2\,\sigma_{x\,y}+c_{2}\right)}{\left(\mu_{x}^{2}+\mu_{y}^{2}+c_{1}\right)\,\left(\sigma_{x}^{2}+\sigma_{y}^{2}+c_{2}\right)},$$

where μ_*x*_ and μ_*y*_ are the average values, $\sigma_{x}^{2}$ and $\,\sigma_{y}^{2}$ are the variances, σ_*xy*_ is the covariance of *x* and *y*, *c*_1_ = (*k*_1_*L*)^2^ and *c*_2_ = (*k*_2_*L*)^2^ are two variables where *L* is the dynamic range of the pixels (here 1 for normalized PSD), and *k*_1_ = 0.01 and *k*_2_ = 0.03 by default.

#### Pearson’s Correlation Coefficients (CORR)

CORR is defined by Eq. (9), where A and B represent 2D matrices;$\bar{A}$ and $\bar{B}$ represent their respective average values and; *i* and *j* are the rows and columns of the matrices (Pearson, 1896).

(9) $$C\,O\,R\,R=\frac{\sum_{i}\sum_{j}\left(A_{i\,j}-\bar{A}\right)\,\left(B_{i\,j}-\bar{B}\right)}{\sqrt{\left(\sum_{i}\sum_{j}{\left(A_{i\,j}-\bar{A}\right)}^{2}\right)\,\left(\sum_{i}\sum_{j}{\left(B_{i\,j}-\bar{B}\right)}^{2}\right)}}$$

### Performance Assessment

#### Clinical Annotation

From the 30-s data, the longest episode that contains at least one localized stable “rotor” (a “rotor” being defined as a series of PSs detected at a “similar” location across subsequent frames over time. – please see section “Rotor Identification From Detected PSs” for a more detailed discussion on PSs and rotors) was selected visually, by an expert, for each patient. The time of the appearance and disappearance of the rotors were used as starting and ending points of the segments. A total of 10 phase episodes of localized stable “rotors” were selected (394.7 ± 59.2 ms), and all PSs were identified frame-independently as “gold standard.” All PSs occurring inside the defined segments were visually annotated, independently of being the longest rotor or not, by an expert. These locations of PSs were considered as the “gold standard” for measuring the performance of all algorithms. The performance of PS detection from all algorithms were compared with this “gold standard” (Supplementary Material and Supplementary Videos. In the videos, the red dots refer to manually annotated PSs of the stable rotor, based on which the episodes were selected. The white dots refer to manually annotated PSs elsewhere during the rotor lifespan).

#### Definition of True/False Positives/Negatives

The PS detections were applied on the 2048-channel maps, with each channel associated with a unique node from the mesh – which can be either a 2D uniform rectangular projected mesh or a 3D triangular mesh representing true LA geometry (Figure 1B). For each frame, we have tested each node on the 2048 mesh, whether this node has been identified as PS or not, and a true positive (TP) value was defined in case an automatically identified PS was within a pre-defined tolerance of 5 mm from a manually annotated PS. The choice of this 5 mm tolerance was defined considering that catheter ablation usually creates a lesion size from 6 to 9 mm (Wittkampf and Nakagawa, 2006). The average inter-electrode distance of the VEGMs is around 3–4 mm, hence the error of detection for 5 mm distance would represent no more than the averaged one-node distance. If more than one PS were detected by the algorithms referring to the same manually annotated PS, false positives (FPs) were recorded. After the TPs and the FPs around the manually annotated PSs were defined, a FP was also recorded if no manually annotated PS were present in regions where the algorithms detected PSs. Similarly, a false negative (FN) was recorded when no PSs were detected within a distance of 5 mm of the manually annotated PS, and a true negative (TN) was recorded when no PSs were detected within that 5 mm radius.

#### Precision and Recall

Phase maps have been shown to usually contain 1–4 PSs from 2048 nodes during persAF (Dastagir et al., 2016). Such dataset is highly imbalanced with many more data points in negative class than positive class. The commonly used receiver operating characteristic curve is not appropriate for measuring the quality of detector techniques for such highly skewed data (Davis and Goadrich, 2006). Precision-Recall (PR) values were used to assess the algorithms, offering a more informative picture of their performance (Davis and Goadrich, 2006), accordingly (Eq. 10):

$$P\,r\,e\,c\,i\,s\,i\,o\,n=\frac{T\,P}{T\,P+F\,P}$$

(10) $$R\,e\,c\,a\,l\,l=\frac{T\,P}{T\,P+F\,N}$$

#### F1 Score in General Form

PS detection is the first step toward finding a rotor – which is usually defined as a PS that persists for multiple consecutive frames either anchored in a location or meandering within nearby regions (Salinet et al., 2017). The best strategy to accurately characterize a rotor as potential ablation target using PS detection might be decreasing FPs and maximizing TPs. Over-detection (FPs) may be less important than missed-detections (FNs) since PSs are usually checked against a time threshold for rotor identification (see section “Rotor Identification From Detected PSs”) (Rodrigo et al., 2017; Roney et al., 2017; Li et al., 2019). Precision is, therefore, less important than recall for the optimization of the parameters, considering the much higher occurrence of negative values than positive values. Consequently, the general form of the *F*_β_ score formula was used (Eq. 11), where the weight for precision (β) chosen was 2, which weighs recall higher than precision. *F*_β_ scores in such form are used as measures of performance of the algorithms with all possible combinations of parameters.

(11) $$F_{\beta }=\left(1+\beta^{2}\right)\cdot \frac{P\,r\,e\,c\,i\,s\,i\,o\,n\cdot R\,e\,c\,a\,l\,l}{\beta^{2}\cdot P\,r\,e\,c\,i\,s\,i\,o\,n+R\,e\,c\,a\,l\,l}$$

PS detections were performed by the different algorithms using different combinations of the phase gradient threshold (from 0.1π to 2π, with 0.1π step) and the search radii (from 1 to 8 nodes, four kernels for algorithm 3). The optimal parameter settings were found by maximizing the *F*_β_ score in the training set.

#### Cross-Validation

10-fold cross-validation was used to test the performance of the binary classifiers/detectors, to minimize the effect of over-fitting from limited data samples. For each iteration, data were divided into training set and testing set. We have tested all possible parameter combinations with the phase gradient thresholds ranging from 0.1π to 2π and the search radii varying from 1 to 8 nodal distances (four kernels for algorithm 3). The parameter settings of the maximum *F*_β_ score generated from all the training sets were selected and tested in the testing set (Supplementary Figure S1).

### Processing Time

Processing times for the algorithms using default threshold and different search radii were measured using MATLAB (R2018a). A desktop PC running 64-bit Windows 10 professional (Microsoft, Redmond, WA, United States, Intel Xeon CPU E5-1630 v4 @ 3.70 GHz quad-core processor with 32 GB DDR4 RAM) was used to test the processing speed in all cases.

### Statistical Analysis

All data are presented as average value and standard deviation. Ordinary one-way ANOVA test was performed for the processing time comparisons. *P*-value lower than 0.05 was considered statistically significant.

## Results

### Agreement Between Automated PS Detection Algorithms

**FIGURE 3:**
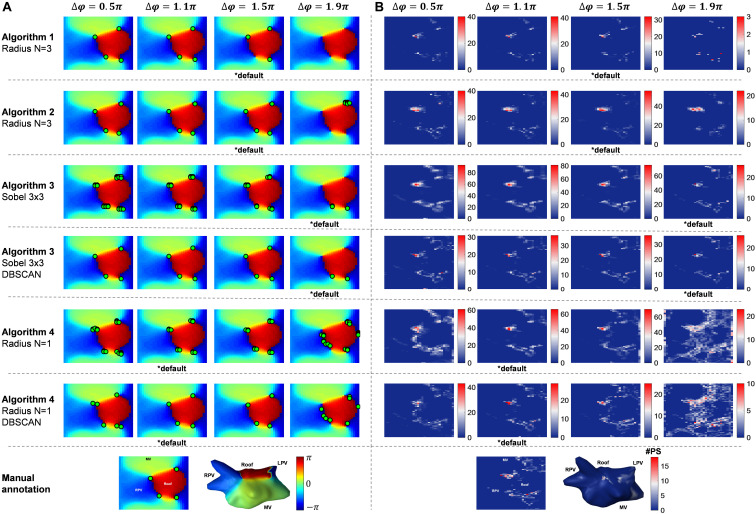
The effect of different phase gradient thresholds. **(A)** An example of the performance of the Algorithms 1–4 and Algorithm 3 and 4 with DBSCAN different phase gradient thresholds, the bottom row is the 3D and 2D phase map with manual annotation. **(B)** PSD maps of the example VEGMs (476.5 ms) using algorithms with different phase gradient thresholds, the bottom row is the 3D and 2D PSD maps with manual annotation.

Figure 3A illustrates the resulting PSs detected by each algorithm using their default thresholds (starred with ^∗^) for both phase gradient and search radius at one time instant. Comparing with the “gold standard” (manual annotation), both under-detection and over-detection can be observed from the resulting maps.

PSD maps (476.5 ms) using default thresholds (starred with ^∗^ in Figure 3B) highlights different accuracy performance when compared with the PSD of the “gold standard.”

The differences in performance using the default parameters in each algorithm are also reflected by the *F*_β_ scores (row 3 in Table 1).

**TABLE 1:** The *F*_β_ scores (accuracy measurement vs. “gold standard”) of each algorithm with their default parameter settings and revised optimal settings.

**Algorithm**	**1**	**2**	**3**	**3 DBSCAN**	**4**	**4 DBSCAN**
**Parameter**						
**Default**						
Phase gradient	1.5π	1.5π	1.9π	1.9π	1.1π	1.1π
N or kernel	3	3	Sobel 3 × 3	Sobel 3 × 3	1	1
*F*_β_	0.527	0.532	0.517	0.524	0.654	0.606
**Optimal**						
Phase gradient	0.8π	0.1π	π	1.9π	1.2π	π
N or kernel	2	3	Nabla 2 × 2	Nabla 3 × 3	1	2
*F*_β_	0.547	0.645	0.742	0.828	0.656	0.831*

N, search radius (# nodes). *Best performance.

**FIGURE 4:**
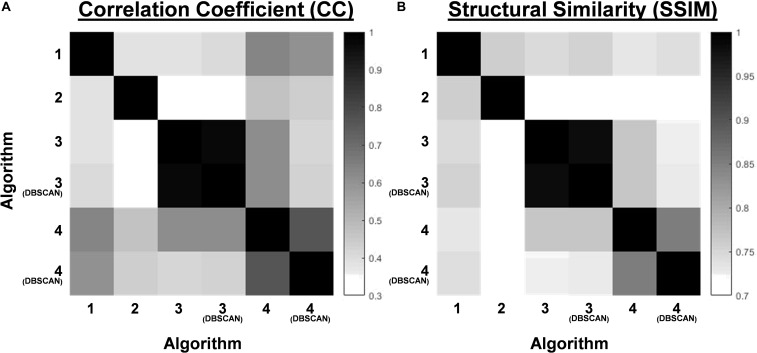
**(A)** The correlation coefficient (CC) of the PSD maps between the Algorithms 1–4 and Algorithm 3 and 4 with DBSCAN based on default parameter settings. **(B)** The Structural Similarity Index (SSIM) of the PSD maps between the Algorithms 1–4 and Algorithm 3 and 4 with DBSCAN based on default parameter settings.

SSIM and CORR were measured and compared between PSD maps created by each algorithm using their default settings for all patients, and were found to have relatively low agreement between each other – except algorithms 3 and 4 and their respective application of DBSCAN clustering (Figure 4).

### Phase Gradient Threshold

The average node distance for all patients was 3.45 ± 2.03 mm. Search radius was defined as *N* = 3 (nodes) by default for algorithms 1 and 2, not applicable for algorithm 3, and *N* = 1 for algorithm 4. Figure 3A shows the phase maps at one time instant and PS detections from the algorithms using 0.5π, 1.1π, 1.5π, and 1.9π as phase gradient thresholds, respectively. Different phase gradient thresholds resulted in different PS concentrations as illustrated by the PSD maps in Figure 3B. Consequently, each method – and their respective thresholds – annotated distinct LA regions as potential targets for ablation.

**FIGURE 5:**
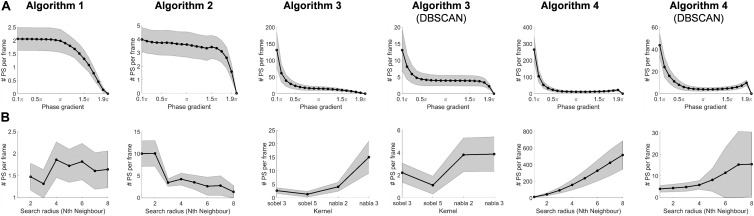
**(A)** The effect on the number of detected PSs by changing the phase gradient thresholds. **(B)** The effect on the number of detected PSs by changing the search radius (kernels in Algorithm 3).

Figure 5A highlights the effect of different phase gradient thresholds on the number of PSs per frame for each algorithm. As expected, the number of PSs per frame decreases with the increase of the threshold.

### Search Radius

**FIGURE 6:**
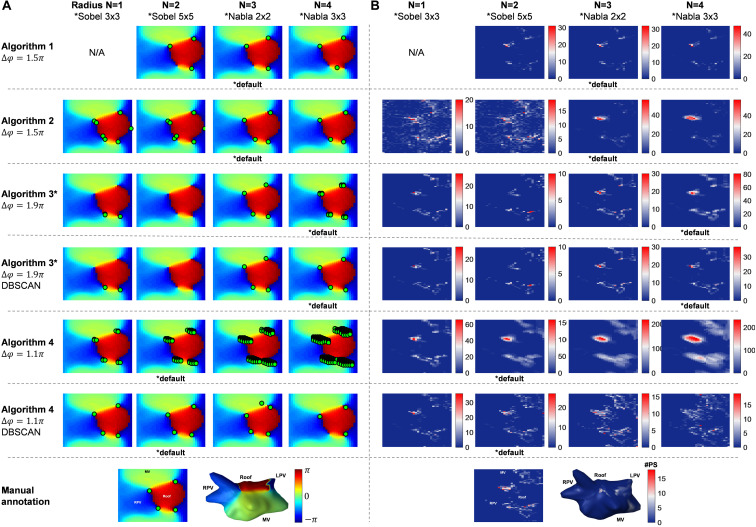
The effect of different choice of search radius (kernels in Algorithm 3). **(A)** An example of the performance of the Algorithms 1–4 and Algorithm 3 and 4 with DBSCAN different search radius parameter, the bottom row is the 3D and 2D phase map with manual annotation. **(B)** PSD maps of the example VEGMs (476.5 ms) using algorithms with different search radius parameter, the bottom row is the 3D and 2D PSD maps with manual annotation.

Similarly, Figure 5B illustrates the effect of adjusting the search radius – or kernel types – on the number of PSs per frame for each algorithm, with different behaviors. Figure 6A illustrates an example of a phase map with the detections performed by the different algorithms using their respective default phase gradient thresholds. Figure 6B shows their respective PSD maps, demonstrating the effect of changing the search radius on the number of PSs per frame for algorithms 1, 2 and 4, and the effect of different convolutional kernels for algorithm 3. While algorithm 1 showed relatively small changes, algorithm 2 was more sensitive to different search radii. Algorithm 4 was the most sensitive to different search radii, producing more over-detections with larger search radius. The DBSCAN clustering step in algorithms 3 and 4 improved the results.

### Processing Time

**FIGURE 7:**
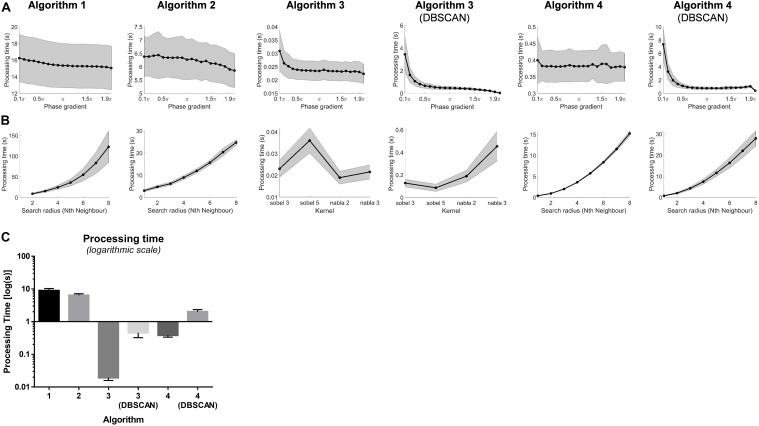
**(A)** Processing time of the PS detection by changing the phase gradient thresholds. **(B)** Processing time of the PS detection of different search radius. **(C)** Processing time (mean and standard deviation) of PS detections using the Algorithms 1–4 and Algorithm 3 and 4 with DBSCAN with optimal thresholds.

Figure 7A illustrates the behavior of the processing time of all algorithms varying the phase gradient thresholds. The processing time decreased with higher phase gradient thresholds, especially for the algorithms with clustering steps (algorithms 1, 2, 3 + DBSCAN, 4 + DBSCAN).

Figure 7B illustrates the processing time of all algorithms with search radius up to 8 circles of neighbors around the points of interest. Except for algorithm 3 and 3 + DBSCAN with kernels, the processing time increased with when more neighbors were included – with a power-law-like behavior for algorithms 1, 4, and 4 + DBSCAN.

The overall processing time for PS detection for an average of 394.7 ms long 2048-channel VEGMs using optimal thresholds for algorithms 1, 2, 3, 3 + DBSCAN, 4 and 4 + DBSCAN were 8.9 ± 1.4 s, 6.4 ± 0.7 s, 0.02 ± 0.003 s, 0.45 ± 0.13 s, 0.38 ± 0.05 s and 2.0 ± 0.3 s, respectively (*p* < 0.0001, Figure 7C).

### Performance Assessment

**FIGURE 8:**
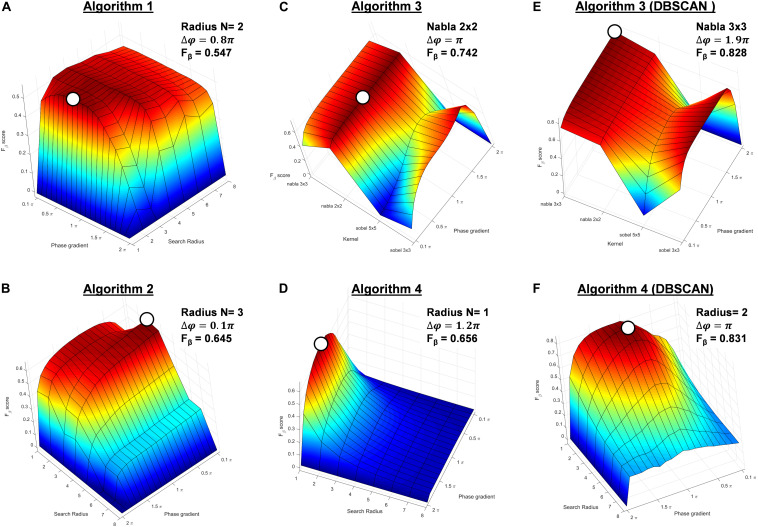
The surface and line plots of *F*_β_ score of the testing data sets of all possible combinations of phase gradient and search radius (kernels for Algorithm 3 and Algorithm 3 + DBSCAN) thresholds of **(A)** Algorithm 1, **(B)** Algorithm 2, **(C)** Algorithm 3, and **(D)** Algorithm 3 + DBSCAN, **(E)** Algorithm 4, and **(F)** Algorithm 4 + DBSCAN (optimal settings regarding each metric highlighted as with circle).

In Figure 8, the colors on the 3D surface color-coded maps represent the *F*_β_ scores of the testing data sets of all possible parameters for all algorithms. The setting with maximum *F*_β_ score was considered as optimal (Table 1). With optimized settings, *F*_β_ score for algorithm 1 increased from 0.527 to 0.547; for algorithm 2, from 0.532 to 0.645; for algorithm 3, from 0.517 to 0.742; for algorithm 3 + DBSCAN, from 0.524 to 0.828; for algorithm 4, from 0.654 to 0.656; and for algorithm 4 + DBSCAN, from 0.606 to 0.831.

Algorithm 4 + DBSCAN clustering showed the best performance over the algorithms according to the *F*_β_ score.

## Discussion

In the present work, we compared four computer algorithms for automated PS identification from phase maps calculated from high-density NCM during human persAF. Two important parameters commonly used for PS detection were investigated: (i) the phase gradient threshold for the dispersion of phase values around points of interest and; (ii) the searching radius, i.e., the number of direct neighbors to be included for the phase gradient probing (different kernels for algorithm 3). Our results show that AF driver identification is dependent on the PS detection algorithm and their parameters – the phase gradient and the search radius. Accordingly, different parameters applied by different research groups would result in distinct AF driver detection, which could explain inconsistencies in rotor-guided ablation outcomes in recent investigations (Benharash et al., 2015; Buch et al., 2016; Gianni et al., 2016; Steinberg et al., 2017). Additionally, our results suggest that the algorithm that best performs for real-time automated PS detection is based on topological charge from 3D triangular meshes with additional spatial clustering. Interestingly, topological charge using convolutional kernel and further spatial clustering has also shown best results for 2D uniform rectangular meshes. Those two algorithms resulted in best performance and the fastest computational speed highlighting their potential use in real-time EP studies. Such algorithms – and their respective optimal parameters – should be considered in future clinical studies for the identification of AF drivers in order to minimize methodological heterogeneities.

### Phase Mapping Using NCM

Previous studies showed moderate correlation between non-contact and contact mapping (Schilling et al., 2000; Earley et al., 2006; Jarman et al., 2012). Schilling et al. (2000) found a correlation of 0.74 ± 0.19 for 3600 electrograms tested in the right atrium; Earley et al. (2006) showed similar correlation 0.81 (0.27–0.98) from the LA; Jarman et al. (2012) showed a correlation of 0.7 ± 0.15 for 62 random locations in the LA; finally, it was also shown that correlation decreased with increasing distance between the endocardial node and the balloon (Schilling et al., 1998; Thiagalingam et al., 2004; Earley et al., 2006). These comparisons, however, were limited on the correlation of the electrograms’ morphology. The use of NCM in the frequency domain was validated by Gojraty et al. (2009) where no significant difference was found in the mean DFs between contact and non-contact signals. Recently, we have shown co-localized behaviors of high frequency sites and PSs in humans (Salinet et al., 2017), suggesting that non-contact phase mapping could be a reliable technique to investigate pro-arrhythmic re-entrant activity, supporting the concept of rotors co-existing with high frequency in isolated sheep hearts (Mandapati et al., 2000).

Roney et al. (2017) have recently suggested the accuracy of PS detection might be dependent on the spatial resolution of the atrial map (i.e., the inter-electrode distance). The authors also concluded that the inter-electrode distance should not be higher than 14.2 mm for a robust phase analysis. Interestingly, 12.6% of the inter-electrode distances in the 64-electrode global basket catheter commonly used during focal impulse and rotor modulation (FIRM) mapping were >20 mm, suggesting these leads could be prone to false PS detections (Roney et al., 2017).

NCM provides an interesting solution for phase mapping by providing high-density simultaneous panoramic atrial coverage and 3D geometry. It provides up to 2048 measuring points in the atrium – resulting in an average node distance of 3.45 mm in the present cohort. The 2048 VEGMs, however, are a result of numerical computation from the non-contact 64 physical electrodes, which may share similar limitations with the 64-electrode contact basket. Further validation of phase mapping using different inter-electrode distances for NCM should be performed in future studies.

When considering the robustness of the algorithms with different spatial resolution, algorithm 4 + DBSCAN is less affected by changing the search radius from 1 to 4 (Figure 5B). This suggests that algorithm 4 would be able to provide accurate detection from 3.45 mm (search radius = 1) to 13.8 mm (search radius = 4), in line with recent findings (Roney et al., 2017).

### Pre-processing of Phase Mapping

Different methods can be considered for generating instantaneous phase signals from time series data – such as the VEGMs (Gray et al., 1998; Umapathy et al., 2010). One of the methods extracts instantaneous phase of the signal from phase-state plots created with delayed versions of the original signal, which requires a judicial choice of the delay (Gray et al., 1998; Umapathy et al., 2010). Hilbert transform provides a solution for generating a phase-shifted signal without the need to choosing a delay. This made Hilbert transform a popular choice when computing instantaneous phase (Bray and Wikswo, 2002a; Nash et al., 2006; Umapathy et al., 2010). Signal processing algorithms have been applied on intracardiac signals prior to Hilbert transform – and consequently phase mapping – to “unmask” the rotary behaviors within narrower frequency ranges. These include wavelet/sinusoidal reconstruction and band-pass filters centered at DFs to filter out unwanted and/or non-physiologic activations (Rodrigo et al., 2014; Kuklik et al., 2015). In addition, further spatial filtering was shown to reduce noise and increase accuracy in sparse grids (Gurevich and Grigoriev, 2019). Naturally, different processing steps prior to the phase mapping may result in different phase maps. Wavelet/sinusoidal reconstruction (Kuklik et al., 2015) was frequently used in intracardiac electrograms, which has been reported to produce comparable results as the FIRM mapping (Alhusseini et al., 2017) and local activation maps (Podziemski et al., 2018). Therefore, wavelet/sinusoidal reconstruction has been chosen for NCM processing in the present study (Kuklik et al., 2015). However, a less aggressive wider band pass filter could be preferred considering the turbulent nature of persAF that results in unstable DF over time. NCM considers an inverse-solution that can smooth the estimated intracardiac signals and generate more sinusoidal-like unipolar VEGMs. The effect of such “strong” filtering/reconstruction steps should be investigated in NCM, which is out of the scope of the current study.

### Optimized PS Detection

Different methods for automated PS detection have been proposed and have been broadly used in EP studies, each of which considering different aspects and characteristics of the phase map (Bray et al., 2001; Rantner et al., 2007; Tomii et al., 2015). In the present study, we have demonstrated that automated PS detection – and consequently ablation target identification – vary significantly for the same individual, depending on the method being used and parameters being applied. We propose revised parameters that optimize the PS detection performed by the different algorithms according to a clinical “gold standard.”

In the present study, the best *F*_β_ score among all algorithms using their respective optimal parameters was 0.831. The optimized parameters resulted in lower phase gradient thresholds comparing to the default parameters for most algorithms, indicating that default thresholds might have been over-estimated, which might contribute in generating a discontinuity in PSs tracking across different time frames. This could impose limitations especially when rotor duration is defined as a key parameter for defining ablation targets (Narayan et al., 2012a,b; Zaman et al., 2018). A lowered and optimized phase gradient threshold could generate a cluster of over-detected points referring to the same PS. With additional spatial clustering method, the over-detected PSs could be easily refined and replaced by the one PS in the cluster with greatest phase gradient. This could be beneficial, as it will minimize the chances of causing discontinued PSs across time.

All algorithms demonstrated value ranges for phase gradient that generated a flat PS detection (Figure 5A). This suggests the algorithms might be robust if the optimal threshold lies in the region of flat detection – where performance is less sensitive to the choice of parameter. Algorithms 3, 3 + DBSCAN, 4 and 4 + DBSCAN showed a faster coverage to a relatively stable region of the curve, demonstrating they could be more robust to be used on different datasets.

### Rotor Identification From Detected PSs

Rotor-guided ablation has become an important topic in AF treatment (Narayan et al., 2012a,b). While early data helped to consolidate rotor-guided ablation as a promising therapy for persAF (Narayan et al., 2012a,b), more recent works have failed to reproduce such promising results (Benharash et al., 2015; Buch et al., 2016; Gianni et al., 2016; Steinberg et al., 2017). While a PS is defined as a “phase discontinuity” around which the phase changes over 2⁢π in a single frame, a rotor is described as a series of PSs detected at a “similar” location across subsequent frames over time. Therefore, the identification of PSs represents a crucial step for the detection of rotors – and consequently AF drivers – during EP studies (Clayton and Nash, 2015; Kuklik et al., 2017). Usually, PSs are detected from a single frame, whilst a rotor is associated with a PS that persists for multiple consecutive frames either anchored in a location or meandering within nearby regions, both which consider a given spatial threshold (Salinet et al., 2017). There is, however, little literature regarding how different research groups define this spatial threshold. Spatial threshold can be defined based on different criteria, such as 1) fixed threshold on distance between the PS first appearance to find stable rotors; and 2) fixed threshold on the distance between consecutive frames, which allows the rotor to drift along (Li et al., 2017a). Meandering rotors were recently reported by our group using NCM in humans (Li et al., 2017a; Salinet et al., 2017). In such cases, a robust tracking method would help to distinguish different types of rotors, and different ablation strategies could be delineated according to the spatial stability and size of the rotor. Such strategy might include the decision whether to ablate at the core of the rotor or to create lines for objecting the wave front propagation around the rotor.

Similarly, the temporal stability is another important feature of a “rotor.” Even though there is no unified definition of a “rotor,” it is usually the case that the core of the rotor needs to stay anchored in a location for a certain duration, in order to be considered as a “true” re-entry circuit (Narayan et al., 2012b, 2014). Two forms of temporal measurement are usually adopted when assessing PSs in subsequent frames during rotor classification: (1) completeness of rotation, i.e., a rotor is defined when one or two full circles of movement are observed (Narayan et al., 2012b) and; (2) duration thresholding, i.e., a PS should exist for a minimum duration (subsequent frames) to be considered a rotor (Rodrigo et al., 2017; Li et al., 2019). However, it is not fully known whether the rotational characteristics of such “rotors” are directly related to AF drivers. These would require prospective studies and the confirmation from ablation strategies targeting such regions to validate their relevance. Whilst still a subject under debate, there are reports on “rotors” with turns of less than 360° that may represent relevant substrate features (Allessie et al., 2010; de Groot et al., 2010; Haissaguerre et al., 2013). The rotors found in the present cohort were not spatially stable. On the contrary, they drifted to different regions of the left atrium (Supplementary Videos). The longest rotor lasted for 460 ms, and the average duration of the rotors were 394.73 ± 59.23 ms. These observations might not be considered rotors if a stricter definition is applied (e.g., with a full “turn” or longer than 1 s).

The present work helps to objectively outline a universal definition of PSs during human persAF, which could prove crucial for comparing rotor-guided ablation outcomes amongst different research/clinical centers.

### Processing Time

Novel computer algorithms for AF driver identification – and consequently targets for ablation – have been extensively explored to study the underlying persAF mechanisms aiming to improving ablative treatment outcomes (Narayan et al., 2014; Li et al., 2017b; Rios-Munoz et al., 2018). Real-time implementation of rotor detection has shown great potential (Rios-Munoz et al., 2018), hence the investigation of the processing time is important for the further development of real-time EP tools to guide catheter ablation of AF. Our results show the convolutional kernel method (Algorithm 3) was faster than the neighbor-indexing algorithms (algorithms 1 and 2) – in which the latter needed a larger number of loop operations for checking the monotonic increase/decrease in phase values in loops of neighbors. Algorithm 4 has shown to have reasonable processing time and was faster than algorithms 1 and 2, as fewer loops were used in counting the “phase jump” comparing to checking monotonic increase/decrease.

DBSCAN has shown to increase the processing time in algorithm 3 and 4, and the choice parameters could influence the processing time of DBSCAN steps – as it is expected that more PS candidates will result in longer clustering time. Therefore, an optimal set of parameter setting would benefit the application of automated PS detection methods in real-time EP studies with minimal increase in procedure time.

### Limitations

The current study was conducted with a relatively small number of patients. *In vivo* data was analyzed retrospectively, which hinders the identification of the “ground truth” for rotor-based AF perpetuation. Nevertheless, the visual annotation performed by a specialist provides a clinically driven “gold standard.” Further investigations using computer models, in which the “ground truth” is known, would be helpful to validate the recommended thresholds (Grandi et al., 2011), but since the end application is for performing AF ablation in humans, the approach taken here is somehow justified.

Not all PS detection algorithms were included in the comparison (Tomii et al., 2015; Lee et al., 2016). Visual annotation of stable rotary PS episodes used as a “gold standard” for assessing performance ensured the true existence of rotational behaviors but may have introduced a further degree of subjectivity in the current study which should be avoided. A more accurate annotated PS database may help to improve the performance of these algorithms. Manual identification of PS points, frame-by-frame is rather time-consuming, so only part of the full data length was manually annotated and used in this study.

## Conclusion

In the present study, we demonstrate that automated PS detection – and consequently persAF ablation target identification – vary significantly for the same individual, depending on the method being used and parameters being applied. We propose revised parameters that optimize the PS detection performed by the different algorithms according to a clinical “gold standard.” Four algorithms were evaluated – a 2D image node-neighbor; a 3D node-neighbor; a 2D convolutional kernel topological charge; and a 3D topological charge. Optimal parameters were proposed for each algorithm and should be used in future studies to improve the accuracy of PS detection. The 3D topological charge with DBSCAN clustering and proposed parameters has shown the best accuracy. Similarly, the algorithm that estimates topological charge using a convolutional kernel with DBSCAN clustering and proposed parameters should be preferred for uniformed 2D meshes. The present study represents a step toward a unified definition/algorithm of phase-derived PS detection with standardized gradient and spatial thresholds, which is essential to allow objective comparisons of outcomes of rotor ablation for persAF therapy among different research/clinical centers.

## Data Availability Statement

All datasets generated for this study are included in the article/Supplementary Material.

## Ethics Statement

The studies involving human participants were reviewed and approved by the East Midlands – Leicester South Research Ethics Committee. The patients/participants provided their written informed consent to participate in this study.

## Author Contributions

XL contributed to the concept and design study, data analysis and interpretation of results, drafting manuscript, critical revision of manuscript, statistics, and “offline” data collection. TA contributed to the data analysis and interpretation of results, drafting manuscript, critical revision of manuscript, and statistics. ND and MG contributed to the data analysis and interpretation of results, critical revision of manuscript, and statistics. JS contributed to the data analysis and interpretation of results, critical revision of manuscript. GC contributed to the data analysis and interpretation of results, critical revision of manuscript, and “offline” data collection. PS contributed to the EP study, data collection, interpretation of results, and critical revision of manuscript. FS contributed to the concept and design study, data analysis and interpretation of results, critical revision of manuscript. GN contributed to the EP studies and ablation procedures, concept and design study, interpretation of results, and critical revision of manuscript. All authors contributed to the article and approved the submitted version.

## Conflict of Interest

GN received a research fellowship from St. Jude Medical (now Abbott) and speaker fees and honoraria from Biosense Webster. The remaining authors declare that the research was conducted in the absence of any commercial or financial relationships that could be construed as a potential conflict of interest.
