# Supplementary material for: Standardizing Single-Frame Phase Singularity Identification Algorithms and Parameters in Phase Mapping During Human Atrial Fibrillation
Source: Front Physiol. 2020 Jul 21;11:869. doi: 10.3389/fphys.2020.00869 (PMC7386053; doi:10.3389/fphys.2020.00869)
Supplement: Supplementary file 1 [file Data_Sheet_1.docx]

Supplementary Material:

Standardizing single-frame phase singularity identification algorithms and parameters in phase mapping during human atrial fibrillation

Xin Li*, PhD^1,2^; Tiago P. Almeida*, PhD^1,2,4^; Nawshin Dastagir, PhD^2^; María S. Guillem, PhD^3^; João Salinet, PhD^5^; Gavin S. Chu, MB BChir, MA(Cantab), MRCP(UK)^1^; Peter J. Stafford, MB BS, MD, FRCP^6^; Fernando S. Schlindwein**, PhD, DSc^2,6^; G. André Ng**, MBChB, PhD, FRCP(Glasgow), FRCP, FESC^1,6^

^1^Department of Cardiovascular Science, University of Leicester, Leicester, United Kingdom

^2^School of Engineering, University of Leicester, Leicester, United Kingdom

***^3^***Universitat Politécnica de València, Valencia, Spain

^4^Aeronautics Institute of Technology, ITA, São José dos Campos, Brazil

^5^Center of Engineering, Modeling and Applied Social Sciences, Federal University of ABC, São Bernardo do Campo, Brazil

^6^National Institute for Health Research Leicester Cardiovascular Biomedical Research Centre, Glenfield Hospital, Leicester, United Kingdom

**Correspondence:**

Dr. Xin Li,

Department of Cardiovascular Sciences/School of Engineering

University of Leicester, LE1 7RH, UK

Tel: +44 (0) 116 229 7380

Email: xl251@leicester.ac.uk

* These authors contributed equally to the manuscript

** These authors contributed equally to the manuscript

# Supplementary Figures and Tables

**Table S1 Clinical characteristics of patients**

| Patient Characteristics  n=10 | | | | |
| --- | --- | --- | --- | --- |
| Male(n) | | 10 | | |
| On amiodarone (n) | | 2 | | |
|  | Median | | Min | Max |
| *Age (years)* | 57.8 | | 36.1 | 76.4 |
| *Days in AF pre-procedure* | 219 | | 132 | 848 |
| *BMI* | 30.4 | | 23.4 | 43.8 |
| *Previous DCCVs* | 2 | | 1 | 5 |
| *Days since last DCCV* | 487 | | 224 | 1237 |
| *Procedure time (min)* | 390 | | 309 | 475 |
| *Fluoro (min)* | 77.2 | | 51.4 | 98.5 |
| *Ablation (min)* | 44.1 | | 26.5 | 109.2 |
| *Ablation area (mm^2^/ %LA)* | 1368 (7.5) | | 627 (3.3) | 2668 (13.6) |

Ten patients undergoing catheter ablation of persAF for the first time were recruited

BMI = body mass index; DCCV= direct current cardioversion.


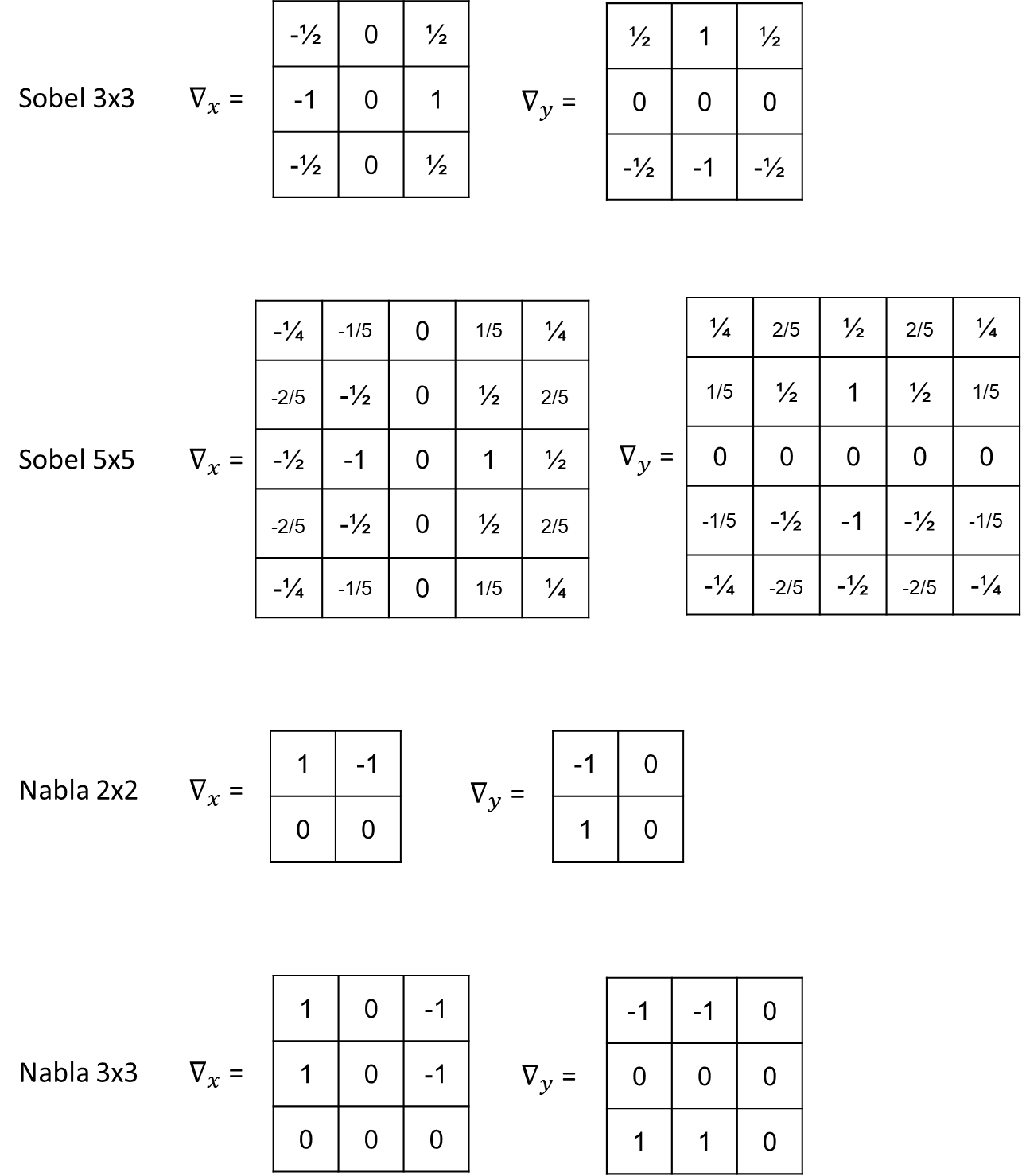


**Figure S1.** Illustration of the four kernels investigated in Algorithm 3


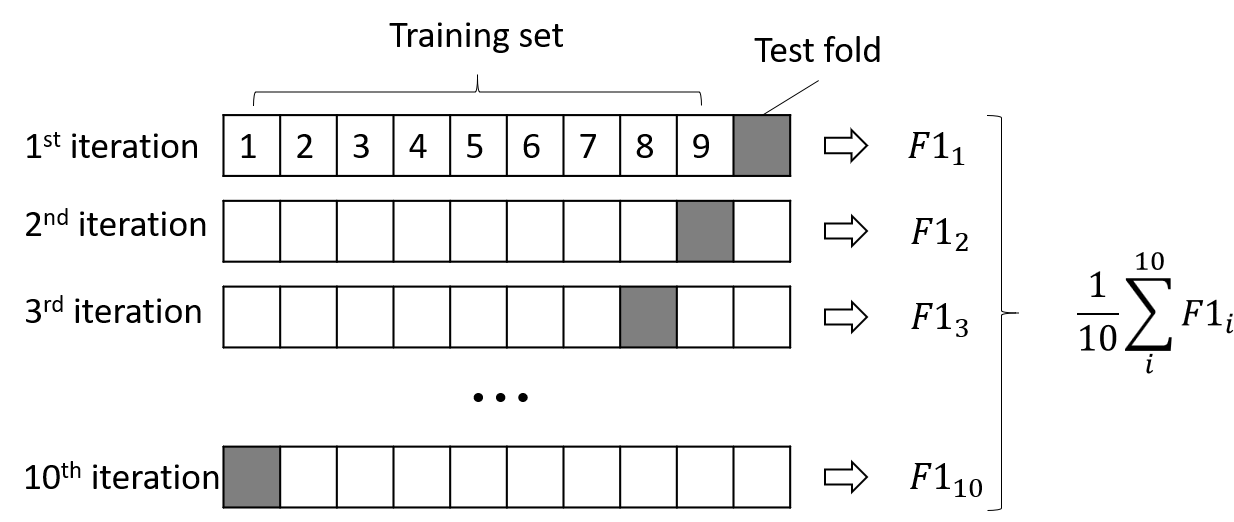


**Figure S2.** Illustration of data partition of the 10-fold cross-validation

**Supplementary Table 2.** The duration of rotor/PS segments with manual annotation

| Rotors | | |
| --- | --- | --- |
| Patient | NO frames | ms |
| 1 | 236 | 460.9 |
| 2 | 199 | 388.6 |
| 3 | 163 | 318.3 |
| 4 | 188 | 367.1 |
| 5 | 211 | 412.1 |
| 6 | 244 | 476.5 |
| 7 | 235 | 458.9 |
| 8 | 203 | 396.4 |
| 9 | 144 | 281.2 |
| 10 | 198 | 386.7 |
| Mean | 202.1 | 394.7 |
| SD | 30.3 | 59.2 |


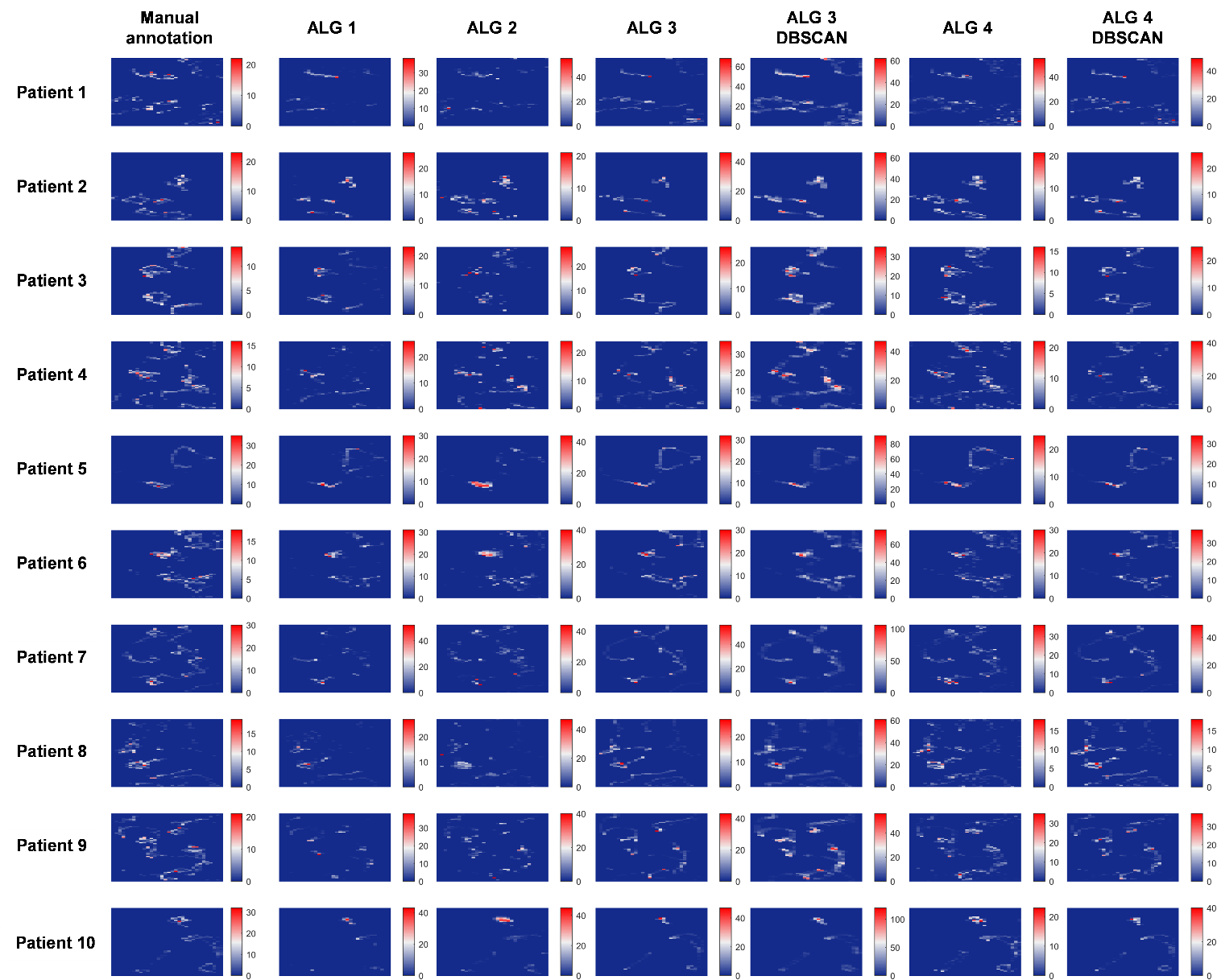


**Figure S3.** PS density maps of the manual annotation and all methods with revised parameters

# Supplementary Videos

| Filenames | Description |
| --- | --- |
| Video x.MP4 | The phase mapping episode selected from patient x. The red dot is the manual annotation of PSs of the ‘stable rotor’ that was visually identified. The white dots are the PSs that were also manually annotated alongside with the red PSs |

x = 1,2,3 …10.
